# Regulation and Sensing of Inflammasomes and Their Impact on Intestinal Health

**DOI:** 10.3390/ijms18112379

**Published:** 2017-11-09

**Authors:** Nicole Ranson, Dale Kunde, Rajaraman Eri

**Affiliations:** School of Health Sciences, University of Tasmania, Launceston, Tasmania 7250, Australia; Nicole.Ranson@utas.edu.au (N.R.); Dale.Kunde@utas.edu.au (D.K.)

**Keywords:** inflammasomes, ulcerative colitis, Crohn’s disease, interleukin (IL)-1β, IL-18

## Abstract

Pattern recognition receptors such as nucleotide-binding oligomerization domain (NOD)-containing protein receptors (NLRs) and the pyrin and hematopoitic interferon-inducible nuclear protein (HIN) domain (PYHIN) receptors initiate the inflammatory response following cell stress or pathogenic challenge. When activated, some of these receptors oligomerize to form the structural backbone of a signalling platform known as an inflammasome. Inflammasomes promote the activation of caspase-1 and the maturation of the proinflammatory cytokines, interleukin (IL)-1β and IL-18. The gut dysregulation of the inflammasome complex is thought to be a contributing factor in the development of inflammatory bowel diseases (IBD), such as ulcerative colitis (UC) and Crohn’s disease (CD). The importance of inflammasomes to intestinal health has been emphasized by various inflammasome-deficient mice in dextran sulphate sodium (DSS) models of intestinal inflammation and by the identification of novel potential candidate genes in population-based human studies. In this review, we summarise the most recent findings with regard to the formation, sensing, and regulation of the inflammasome complex and highlight their importance in maintaining intestinal health.

## 1. Introduction

The gastrointestinal environment is a continuous system with a dual function. Firstly, it provides the human body with the energy it needs to grow and develop and aids in the elimination of waste material. Secondly, it plays an important role in preventing infection by providing a vast array of immune cells close to the mucosal surface to target environment toxins and potential pathogens. Several diseases are known to occur in the gastrointestinal tract; notable examples are ulcerative colitis (UC) and Crohn’s disease (CD). Both are characterised by the chronic and relapsing inflammation of an unknown aetiology. In CD, the inflammation is discontinuous, transmural, and often associated with intestinal wall thickening, ulcerations, bowel strictures, luminal narrowing, and abscesses. While in UC, the inflammation is continuous, usually spreading proximally from the anal verge and affecting only the mucosa and submucosa layers [1]. For both diseases mechanisms that regulate the gastrointestinal innate immune system have been highlighted as contributing to disease pathology.

The innate immune system is the “danger sentinel” of the gut and employs an array of germline-encoded pattern-recognition receptors (PRRs) to continually scan the mucosal environment for signals of host-derived cellular stress or pathogenic microbes. PRRs have the unique ability to remain passive to non-dangerous motifs, such as commensal gut flora, inherent host molecules, or dietary antigens [2]. PRRs primarily recognise conserved microbial molecules known as pathogen-associated molecular patterns (PAMPs) or damage-associated molecular patterns (DAMPs), which are released by the host in response to stress, tissue damage, and necrotic cell death [3,4,5]. Cells involved in first-line defence mechanisms such as dentritic cells, epithelial cells, monocytes, macrophages, and neutrophils are all known to express PRRs [2]. 

Membrane-bound PRRs, such as Toll-like receptors (TLRs) and C-type lectins receptors (CLRs), are responsible for monitoring the extracellular milieu and endosomal compartments of PAMPs and DAMPs, while cytoplasmic surveillance is executed by NOD-like receptors (NLRs), pyrin and HIN domain-containing (PYHIN) family members, RIG-1-like receptors (RLRs), and several cytosolic nucleic acid sensors [6]. Membrane and intracellular receptors work in concert with one another to orchestrate an effective immune response against a pathogenic insult [7]. Often an intact microbial pathogen will contain multiple PAMPs, which trigger the sequential detection by a number of receptors in different subcellular compartments. Upon activation, many of these receptors promote the secretion of proinflammatory cytokines and transcription mediators, and initiate pathways responsible for pathogen neutralisation and elimination [8]. 

Additionally, some NLR receptors and PYHIN receptors are known to form the structural backbone of the multimolecular complex known as the inflammasome. The inflammasome complex is a core component of the inflammatory response and its activation enhances the maturation of pro-interleukin (IL)-1β and proIL-18 to their biologically active IL-1β and IL-18 forms [2]. IL-1β is a multifunctional cytokine and, locally, can induce targets such as IL-6, IL-8, and COX2, stimulate T cell proliferation, and direct neutrophils to the site of injury or infection [4,9,10]. IL-6 can direct the immune response by recruiting monocytes and lymphocytes to replace neutrophils in shifting from innate to adaptive immunity [11].

In the intestine, the inflammasome can also promote an inflammatory form of cell death, known as pyroptosis. Pyroptosis halts the replication of intracellular pathogens by destroying the infected immune cell and exposing the surviving bacteria to circulating phagocytes and neutrophils [12]. Both canonical (caspase-1) and non-canonical (caspase-11) inflammasome pathways are able to induce pyroptosis; however, caspase-11 does not produce mature IL-1β or IL-18. Caspase-11-induced pyroptosis is thought to occur upstream of canonical inflammasomes in response to lipopolysaccharides (LPS) sensed in Gram-negative bacteria. Both mechanisms are considered important for microbial defences in the gut [12,13].

## 2. Formation of a NOD-Like Receptor Protein (NLRP) Inflammasome Complex

**Figure 1:**
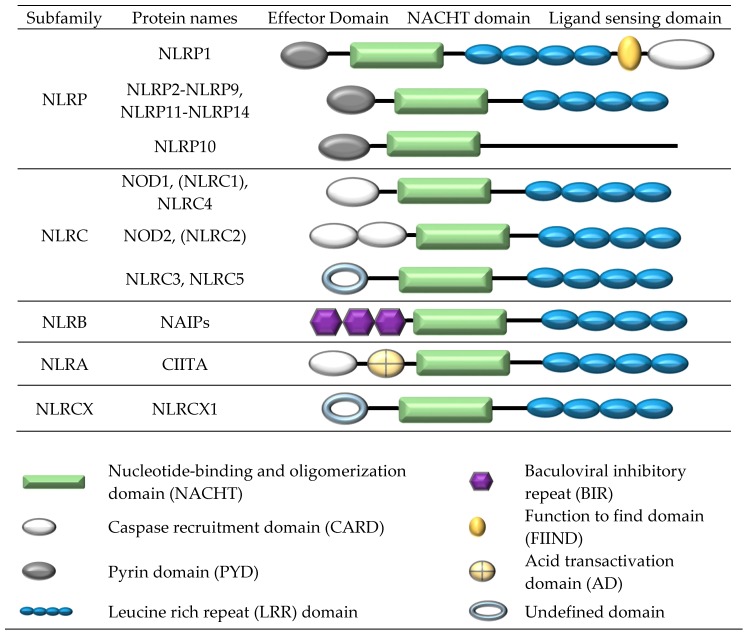
Structure of the human nucleotide-binding oligomerization domain (NOD)-like receptor subgroups.

In general, the NLRP inflammasome complex consists of a nucleotide-binding oligomerization domain (NOD)-like receptor (NLR) protein, a caspase and often an adaptor protein known as apoptosis-associated, speck-like protein containing a CARD (ASC) [2,14]. Several receptors from the NLR family, NLRP1, NLRP2, NLRP3, NLRC4, NLRP6, NLRP7, and NLRP12 (Figure 1), have all shown the ability to form the structural backbone of an inflammasome complex. The ASC adaptor protein is identical for all inflammasomes and contains two transduction domains, a pyrin domain (PYD) domain and a caspase recruitment domain (CARD) domain [15].

**Figure 2:**
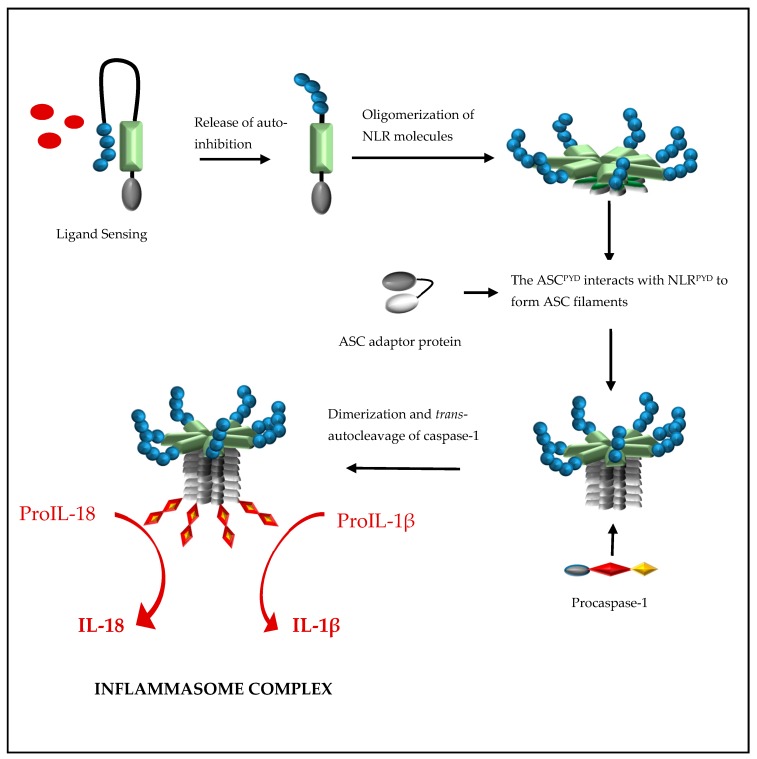
Formation of a NOD-like receptor protein inflammasome containing an N-terminal Pyrin domain.

Formation of a NLRP inflammasome is initiated by ligand activation of the receptor protein and this causes the NLR proteins to oligomerize through their nucleotide-binding and oligomerization (NACHT) domains (Figure 2). This oligomerization creates a platform of NLR^PYD^ molecules at the N-terminal and, through NLR^PYD^/ASC^PYD^ interactions, nucleates helical ASC clusters to form an ASC filament structure. The aggregation of multiple ASC^CARD^ molecules promotes ASC^CARD^/caspase-1^CARD^ interactions, which, in turn, brings caspase domains into close proximity for dimerization, *trans*-autocleavage, and activation [15]. The binding of ASC to both the NLR protein and caspase-1 is facilitated by a 23-residual linker which orientates ASC^PYD^ and ASC^CARD^ back to back, hence preventing steric interference of binding sites while enhancing binding partner prospects [16]. ASC is sequestered in the nucleus but rapidly translocates to the cytoplasm upon stimulation, where it participates in inflammasome formation [17]. Interestingly, inflammasome formation can be abolished by preventing the cellular redistribution of ASC [17].

The NOD-like receptor (NLR) family comprises 23 human members [18,19]. All NLR proteins contain a central nucleotide-binding and oligomerization (NACHT) domain, flanked by a C-terminal LRR domain and N-terminal effector domain. The NACHT domain facilities self-oligomerization and has ATPase activity. The N-terminal domain participates in protein-protein interactions, while the LRR domain is involved in ligand recognition. Subgroup classification is based on the structure of the N-terminal effector region which generally comprises a CARD, PYD, or BIR domain. The NLRP1, NLRP2, NLRP3, NLRC4, NLRP6, NLRP7, and NLRP12 receptors have all shown the ability to form inflammasome complexes.

**Figure 3:**
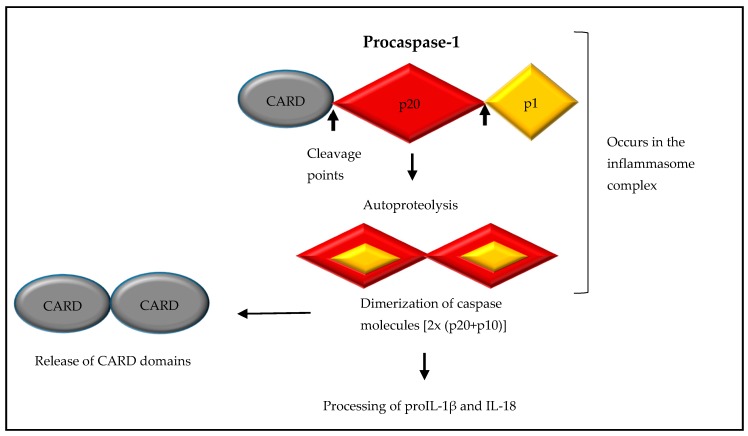
The mechanism for the inflammasome-mediated, catalytic conversion of procaspase-1 to caspase-1.

Caspase-1 is synthesized as an inactive, monomeric zymogen (procaspase-1) and, initially, is cleaved into a p35 fragment containing a CARD and p10 fragment. Autoproteolysis results in the generation of a large p20 subunit and a small p10 subunit, as well as the removal of the N-terminal CARD domain. Dimerization of caspase molecules (p20 and p10) results in the catalytically active caspase-1 enzyme (Figure 3) [20,21]. Inflammasome-activated caspase-1 cleaves its substrates, proIL-1β and proIL-18, at recognition sites adjacent to aspartic acid residues, resulting in mature IL-1β and IL-18 [14].

In contrast to other members of the NLRP subfamily, NLRP1 contains both a function to find (FIIND) and CARD domain at the C-terminal, as well as a PYD domain at the N-terminal [22] (Figure 1). Given that NLRP1 contains two signal transduction domains (PYD and CARD), it can activate caspase-1 through its C-terminal CARD domain without the need for the ASC adaptor protein; however, ASC has been shown to greatly enhance inflammasome formation and IL-1β processing [23]. The FIIND domain is a highly conserved protein region and, based on amino acid sequencing, is only present in two human proteins, NLRP1 and the caspase recruitment domain family, member 8 (CARD8) protein [24]. CARD8 is thought to function as an adaptor molecule that negatively regulates NF-κB activation, caspase-1-dependent IL-1β secretion, and apoptosis, and is often overexpressed in many types of cancers [25,26,27].

NLRP1 inflammasome formation is strictly dependent on autolytic proteolysis within the FIIND domain, and after cleavage the two fragments remain associated to form a processed NLRP1. Dimers of ASC joined by ASC^PYD^/ASC^PYD^ are recruited to the C-terminal NLR^CARD^ domain and bind via NLR^CARD^/ASC^CARD^ interactions. This is in contrast to other NLRP proteins, which recruit ASC to the N-terminal PYD domain and bind via NLR^PYD^/ASC^PYD^ Interactions to form the inflammasome complex. Subsequently, caspase-1, through its CARD domain, interacts with ASC^CARD^, which leads to dimerization, *trans*-autocleavage, and activation of caspase-1 and IL-1β, IL-18 processing [28]. The formation of ASC filaments in the activation of the NLRP1 inflammasome remains to be defined.

Formation of a nucleotide-binding oligomerization domain (NOD)-like receptor protein (NLR) inflammasome is initiated by ligand activation of the NLR protein. This causes the NLR proteins to oligomerize through their NACHT domains to create a platform of NLR^PYD^ molecules at the N-terminal and, through NLR^PYD^/ASC^PYD^ interactions, to nucleate helical ASC clusters to form a filament ASC structure. The aggregation of multiple ASC^CARD^ molecules promotes ASC^CARD^/caspase-1^CARD^ interactions, which in turn brings caspase domains into close proximity for dimerization, *trans*-autocleavage, activation, and the processing of pro-interleukin (IL)-1β and proIL-18 to their biologically active forms, IL-1β and IL-18, respectively.

Caspase-1 is initially synthesized as the inactive monomeric zymogen, procaspase-1. Binding of the procaspase-1^CARD^ to ASC^CARD^ filaments on the inflammasome complex results in the cleavage of procaspase-1 into a p35 fragment containing a CARD and a p10 fragment. Dimerization of the p10 and p20, and the removal of the procaspase-1^CARD^ domain, produces catalytically active caspase-1.

## 3. Structure and Formation of a Pyrin and HIN Domain (PYHIN) Inflammasome Complex

**Figure 4:**
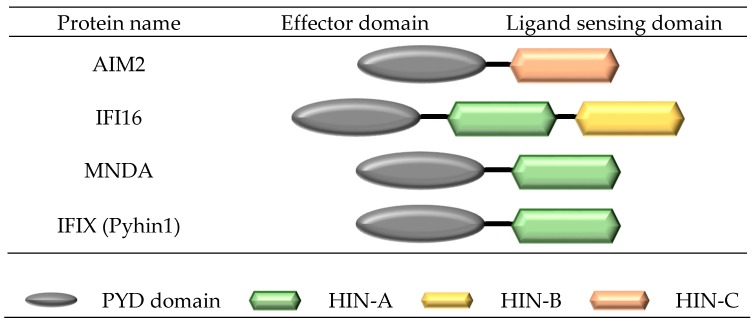
Structure of the human pyrin and hematopoitic interferon-inducible nuclear protein (HIN) domain (PYHIN) family.

Two receptors in the pyrin and HIN domain (PYHIN) receptor family, absent in melanoma 2 (AIM2) and interferon-inducible protein 16 (IFI16), have shown the ability to form inflammasome complexes (Figure 4). Similar to NLRP inflammasomes, PYHIN inflammasomes, such as AIM2, upon ligand activation, oligomerize through their PYD domains to form a platform of AIM2^PYD^ molecules, which preferentially associate with ASC^PYD^ to form ASC filaments. The flexibly linked ASC^CARD^ clusters along the ASC^PYD^ to form a platform for the binding of caspase-1^CARD^. Similar to other NLRP inflammasomes, the ASC filament structure forms the main body of the inflammasome. The interaction of ASC^CARD^/caspase-1^CARD^ brings caspase domains into close proximity for dimerization, *trans*-autocleavage, and activation, and the subsequent maturation of IL-1β and IL-18 [15].

The human pyrin and HIN domain (PYHIN) family of receptors comprises 4 members, including the interferon-inducible protein 16 (IFI16), absent in melanoma 2 (AIM2), myeloid nuclear differentiation antigen (MNDA), and interferon-inducible protein X [IFIX (Pyhin1)], while mouse contains 11 confirmed members [29]. All members consist of an N-terminal pyrin domain (PYD) domain attached to one or more hemopoietic interferon-inducible nuclear protein (HIN-200) domains at the C-terminal. Three distinct forms of HIN-200 have been characterised (HIN-A, -B and -C) and are classified according to specific consensus motifs [30].

## 4. Ligand Sensing of Inflammasome Complexes

Depending on the type of receptor protein in the complex, inflammasomes have the ability to respond to a wide array of pathogens and cellular danger signals. The LRR domains of the NLRP receptors and the HIN200 domains of the PYHIN receptors are thought to be involved in ligand interactions; however, direct binding of an activating ligand to a receptor has only been demonstrated for the AIM2 and IFI16 inflammasomes. 

## 5. The NLRP1 Inflammasome

The NLRP1 inflammasome was one of the first inflammasomes to be described; however, efforts to unravel the processes that lead to activation have been hampered by species variations in the *NLRP1* gene. In humans, the *NLRP1* gene is singular, while in mice the gene encoding *Nlrp1* is polymorphic with three homologs, *Nlrp1a*, *Nlpr1b*, and *Nlrp1c* [20]. Furthermore, the structure of mouse *Nlrp1* lacks the N-terminal PYD domain found in human NLRP1, and five different strain specific *Nlrp1b* alleles exist in inbred mice [31].

*Nlrp1* is activated mainly by lethal toxin (LeTx) produced by *Bacillus anthracis* with variations in *Nlrp1b*, which provides sensitivity or resistance to the toxin [32]. LeTx is a bipartite toxin consisting of a protective antigen-binding subunit and a catalytic lethal factor moiety. Binding of the protective antigen to anthrax binding sites translocates lethal factor into the host cytosol where it cleaves the N-termini of mitogen-activated protein kinase (MAPK), thereby disrupting cell signalling pathways. Initially, lethal factor blocks cytokine production from numerous cell types, inhibits chemotaxis of neutrophils, induces apoptosis in activated macrophages, and later induces cytokine-independent shock and death [33]. Caspase-1 and IL-1β deficient mice are more susceptible to *B. anthracis* infection, indicating IL-1β production via the NLRP1b inflammasome is more important than ASC-independent pyroptosis in the host protective response to *B. anthracis* [21,33].

More recently, NOD2 has been linked to NLRP1-dependent sensing of MDP and *B. anthracis* in activated cells where it produces a NOD2-NLRP1 inflammasome complex [34]. NOD2 is a known intracellular sensor of MDP and has the ability to contribute to the induction of NF-κB and MAPK transcription factors; however, TLRs are much more effective in triggering these responses [35]. The absence of NOD2 prevents *B. anthracis*-induced IL-1β secretion but has little effect on the transcription of proIL-1β, indicating the importance of the NOD2-NLRP1 association in host defences against *B*. *anthracis* [34]. 

## 6. The NLRP3 Inflammasome

The NLRP3 inflammasome has the ability to activate upon exposure to a wide range of whole pathogens, environmental irritants, and structurally diverse DAMPs and PAMPs [2,3,36].

While the mechanisms are not yet fully understood, it is thought that activation of NLRP3 occurs in response to host-derived factors that are altered by these agents. While several models have been proposed for the activation of NLRP3, none have been found to be unified for all activating agents. The proposed mechanisms include;

1. K^+^ efflux [37]
2. The generation of mitochondrial-derived, reactive oxygen species (mROS) [38]
3. Phagolysosomal destabilisation and the release of cathepsins [39]
4. The release of mitochondrial DNA or the mitochondrial phospholipid cardiolipin [40,41,42]
5. Translocation to the mitochondria [38,43,44]

To add to the controversy, membrane permeation, phagolysosmal destabilisation, mitochondrial damage, and ROS production are all interrelated cellular events making the distinction between bystander and causative activation events complicated.

In resting cells, the basal expression of *NLRP3* is insufficient for inflammasome activation and consequently two signals are required for the activation of the NLRP3 inflammasome [45,46]. The first signal is the NF-κB-mediated transcription of *NLRP3* and *proIL-1β* from stimulation of TLR antagonists or cytokines such as TNF-α and IL-1β. The second signal is the ligand activation step, which culminates in the activation of caspase-1 and the maturation of IL-1β and IL-18 [3,47]. The enhanced effect of guanylate-binding protein (GBP5) on Nlrp3 inflammasome assembly in response to bacteria and soluble-but-not-crystalline inflammasome priming agents raises the possibility of agent-specific cofactors being required for inflammasome activation [48]. 

Particulate matter such as aluminium, silica, monosodium urate (MSU), calcium pyrophosphate dehydrate crystals, cholesterol, and amyloid β enters the cell by means of phagocytosis [39,49,50,51]. The destabilisation of the phagolysosmal membrane and the release of the cysteine protease cathepsins B into the cytosol are thought to also trigger NLRP3. Inhibitors of cathepsins B have been shown to prevent caspase-1 activation induced by *N. gonorrhoeae* [52]. Interestingly, cathepsins-deficient mice show minimal defects in the activation of NLRP3 in response to particulate matter, suggesting other off-target effects may exist [53].

More recently, mitochondrial dysfunction and activation of the NLRP3 inflammasome has been an area of intense research and much speculation. Mitochondrial reactive oxygen species (mROS) are produced in response to cell stresses such as hypoxia, starvation, pathogen infection, and growth factor stimulation or membrane damage [54]. The release of mROS and oxidised mitochondrial DNA have both been shown to activate the NLRP3 inflammasome [40,55]. Interruption of ROS production using inhibitors blocks NLRP3 activation, suggesting ROS production upstream is necessary for NLRP3 activation [55,56,57].

It has been proposed that NLRP3 associates with the mitochondria upon activation [43,55] and, when exposed to non-crystalline activators, recruitment from the cytosol to the mitochondria is mediated by the mitochondrial anti-viral signalling protein (MAVS) [44]. MAVS is also known as a mitochondrial adaptor protein and plays a crucial role in RLR receptor signalling pathways, leading to type 1 IFN induction and NF-κB activation [58]. MAVS is thought to directly associate with the N-terminus of NLRP3 to promote optimal inflammasome formation [44]. Consistent with a role for MAVS in NLRP3 activation, MAVS-deficient mice exposed to dextran sodium sulphate (DSS)-induced colitis fail to upregulate IL-1β [59]. 

Other work on mitochondrial dysfunction has demonstrated a ROS-independent activation of *Nlrp3* induced by the antibiotic linezolid, whereby the mitochondrial specific lipid cardiolipin binds to *Nlrp3*, leading to the maturation of IL-1β [42]. Cardiolipin is a phospholipid exclusively found in the inner mitochondrial membrane of eukaryotic cells. Cardiolipin plays a critical role in the activation of caspase-8 and caspase-3 in the apoptotic cell death pathway, which raises the possibility that the inflammasome pathways are linked to the apoptosis pathways by processes that control mitochondrial homeostasis. 

In addition, agents that induce NLRP3 activation, such as nigericin, have demonstrated an ability to disrupt mitochondrial homeostasis by reducing the intracellular concentration of the coenzyme NAD^+^. Low NAD^+^ inactivates the α-tubulin deacetylase sirtuin 2 (SIRT2) and causes the accumulation of acetylated α-tubulin. Excess acetylated α-tubulin mediates the microtubule transport of mitochondria, which drives the apposition of ASC on the mitochondria to NLRP3 on the endoplasmic reticulum. Microtubule transport of organelles creates optimal sites for signal transduction between ASC and NLRP3, and directs activation of NLRP3. Work using inhibitors of tubulin polymerisation has demonstrated suppression of IL-1β [43].

Early work investigating caspase-1 activation by the NLRP3 inflammasome showed that K^+^ efflux accompanies NLRP3 activation [56,60], and a high extracellular concentration of K^+^ blocks the activation of not only the NLRP3 inflammasome but also the NLRP1, NLRC4, and AIM2 inflammasomes [61,62]. ATP levels have been linked to K^+^ efflux, to such an extent that high extracellular ATP levels engage the ATP-gated purinergic P2X_7_ receptor, promoting the formation of the pannexin-1 pore, which induces K^+^ efflux [47,63]. Previous work by Muῆoz-Planillo [37] has shown that ROS generation, opening of the pannexin-1 pore, and K^+^ efflux all occur upon stimulation with a variety of bacterial pore-forming toxins, nigericin, ATP, and particulate matter. However, in contrast to other work, the permeation of the cell membrane to K^+^ and Na^+^ was found to be the only common step induced by all NLRP3 antagonists and the primary activity that was necessary and sufficient for caspase-1 activation. In addition, cytosolic K^+^ efflux was found to be specific to NLRP3 activation and was shown not to play a role in the activation of AIM2. These results await further clarification by other independent researchers.

## 7. The NLRC4 Inflammasome

The NLRC4 inflammasome has been well characterised in the mouse system and plays an important role in the detection of pathogenic bacteria [20]. The pathogenicity of a bacteria is reliant on functional secretion systems, including the type III and IV, which act as needle-like structures delivering virulent factors into the host’s cytosol. NLRC4 is activated by two critical components of pathogenic bacteria, a sequence motif found in the basal rod components of the type III (T3SS) and IV (T4SS) bacterial secretion systems, and a similar sequence motif found in flagellin, which is a component of their flagellum apparatus [64,65]. NLRC4 has been shown to detect basal rod components in *Salmonella typhimurium*, *Legionella pneumophila*, *Burkholderia pseudomallei*, *Escherichia coli*, *Shingella flexneri*, and *Pseudomonas aeruginosa* [66,67], as well as leaked cytosolic flagellin from *Listeria monocytogenes*, *Salmonella typhimurium*, *Pseudomonas aeruginosa*, and *Legionella pneumophila* [65,68,69].

Activation of the NLRC4 inflammasome involves the initial binding of a receptor protein from the neuronal apoptosis inhibitory protein (NAIP) subfamily of NLRs to the activating ligand. NAIP receptor proteins differ from other NLRs in that they contain multiple BIR domains at the N-terminus instead of a CARD or PYD domain (Figure 1). In humans, only one *NAIP* homolog is expressed, whereas in mice the *NAIP* locus is polymorphic and seven paralogs of *Naip* (*Naip1–Naip7*) exist [67]. Human NAIP and its mouse ortholog, Naip1, recognise cytosolic T3SS needle proteins; Naip2 binds T3SS rod components, while Naip5 and Naip6 bind directly to bacterial flagellin [67,69]. Binding of a NAIP protein to a bacterial motif leads to the formation of the NAIP-NLRC4 inflammasome complex and activation of caspase-1. Interestingly, in human U937 monocyte-derived macrophages, NLRC4 activation does not occur in response to flagellin or T3SS rod protein but occurs in response to the T3SS subunit Cprl from *Chromobacterium violaceum*, which raises the possibility that other accessory proteins may be involved in activation of the human NLRC4 inflammasome [69].

## 8. The AIM2 and IFI16 Inflammasome

Both the cytosolic AIM2 receptor and the nuclear IFI16 receptor directly bind their activating-ligand, double-stranded DNA (dsDNA) via the C terminal HIN200 domain [70,71,72]. Non-sequence-specific binding occurs at multiple sites along the dsDNA and through electrostatic attractions between the positively charged HIN domain residues and the dsDNA sugar phosphate backbone [73]. 

The mechanisms that enable AIM2 and IFI16 to respond to viral, bacterial, mammalian, and synthetic dsDNA while remaining unresponsive to self DNA are still unclear [20,74]. 

Work using *Aim2*-deficient mice has demonstrated an essential role for AIM2 in the recognition of viruses and bacteria by the detection of cytosolic dsDNA. When compared to WT mice, *Aim2^−/−^* mice experience higher mortality rates, higher bacterial load, and decreased production of caspase-1-generated cytokines after infection with *Fransicella tularenis*, suggesting AIM2 is necessary for detection of *Fransicella tularenis* [75,76]. Similarly, mouse macrophages deficient for *Aim2* show an impaired ability to recognise not only *Fransicella tularenis* but also vaccinia virus, with murine cytomegalovirus (mCMV) with only partial recognition of *Listeria monocytogenes* being demonstrated [70,77]. 

## 9. The NLRP6, NLRP7, and NLRP12 Inflammasomes

In addition to the well-known NLRP1, NLRP3, NLRC4, AIM2, and IFI16 inflammasomes, NLRP6, NLRP7, and NLRP12 have shown an ASC-dependent activation of caspase-1. However, the signals that activate the NLRP6 and NLRP12 inflammasomes are yet to be determined [78]. 

Indeed, two independent studies have reported a lack of caspase-1 activation and IL-1β release in Nlpr6-deficient mouse macrophages in response to ATP and LPS, which suggests that the triggers that activate the NLRP6 inflammasome are different to those that activate the NLRP3 inflammasome [79,80]. Recently, NLRP7, which is not expressed in mice, was shown to form an ASC-dependent inflammasome in human macrophages in response to microbial acylated lipopeptides [81]. 

## 10. Regulation of the Inflammasome Complex

The potent inflammatory cytokines, IL-1β and IL-18, and the pyroptosis pathway all have the potential to cause tissue damage and disrupt an effective adaptive immune response. The mechanisms that lead to maturation of IL-1β and IL-18 are tightly controlled at several levels and multiple checkpoints along this process, ensuring response-appropriate levels.

In most cells the basal levels of many of the inflammasome constituents is insufficient for inflammasome formation. Consequently, the expression of the inflammasome components is regulated by NF-κB-induced transcription and requires sensitization by a TLR or CLR ligand or stimulus from cytokine-signalling pathways [3,4]. In contrast to most cytokines, IL-1β and IL-18 are produced as inactive zymogens requiring caspase-1 cleavage between Asp and Ala for maturation [82,83]. The synthesis of precursor cytokines requiring activation prevents aberrant secretion of the leaderless IL-1β and IL-18 cytokines. Serine proteinases, such as cathepsins G, elastase, and, in particular, proteinase 3 found in neutrophils, have also been shown to cleave proIL-1β to active IL-1β, while in monocytes, autocrine production of ATP can activate caspase-1 and cleave proIL-1β, thereby releasing IL-1β by transcription only [84]. It is worth noting that during acute inflammatory conditions, non-canonical maturation of IL-1β can also occur via caspase-11 and the NLRP3 inflammasome [12]. 

## 11. Regulation by Autoinhibition of the Ligand-Sensing Domain

For most of the receptor proteins, autoinhibition of the ligand-sensing domain prevents unproductive intramolecular interactions by providing a tight, on-site repression of the protein in the absence of a suitable activating ligand. For NLRP1, NLRP3, NLRP12, and NLRC4 receptors, autoinhibition is achieved by the association of two chaperone proteins, ubiquitin ligase-associated protein (SGT1) and heat-shock protein 90 (HSP90), to the LRR domain. Upon ligand sensing, SGT1 and HSP90 dissociate, resulting in a conformation change within the protein which favours the recruitment of the ASC adaptor protein [85]. Whether autoinhibition of the sensing region occurs for the NLRP6 protein remains to be determined. For the PHYIN subfamily autoinhibition is provided by the molecular interactions between the PYD and HIN-200 domain, and binding of DNA releases this autoinhibition [73].

## 12. Priming Events That Regulate Activation

Specific priming events are known to regulate the activation of inflammasomes. The K-63-specific deubiquitinating enzyme BRCC3 mediates the deubiquitylation of NLRP3, which has recently been shown to occur in response to pattern recognition receptor stimulation [86]. Similarly, and as mentioned above, GBP5 enhances NLRP3 assembly in response to bacterial but not crystalline agents [48]. A priming event involving the phosphorylation of Ser533 by kinases like PKCδ is necessary before *Salmonella typhimurium* can activate the NLRC4 inflammasome. The phosphorylation of Ser533 is thought to result in a conformation change within the NLRC4 protein [87].

The anti-apoptotic proteins B-cell lymphoma 2 (Bcl-2) and B-cell lymphoma extra-large (Bcl-xl) have been shown to regulate the NLRP1 inflammasome. By associating with NLRP1 via their loop domains, Bcl-2 and Bcl-xl are able to suppress caspase-1 activation and IL-1β processing [88,89]. Similar experiments for NLRP3 in Bcl-2-deficient macrophages have shown more caspase-1 processing, while Bcl-2-overexpressing macrophages demonstrated less caspase-1 processing, suggesting NLRP3 may also be regulated by Bcl-2 protein [40]. 

Activation of the NLRP3 inflammasomes is thought to be influenced by K^+^ levels; indeed, low intracellular K^+^ level enhances caspase-1 activation [56,60]. Similarly, high extracellular K^+^ levels block IL-1β release from NLRC4 and AIM2 inflammasome, suggesting that the regulatory effect of K^+^ may be extended to other inflammasome complexes [75,90]. Interestingly, the level of extracellular K^+^ needed to block IL-1β release for the NLRP3 inflammasome is less than that needed for the NLRC4 or AIM2 complex, while for the NLRP7 inflammasome high K^+^ levels only slightly reduced IL-1β release [81]. More work is needed to exclude off-target effects and to determine the reasons for inflammasome-specific thresholds to K^+^ levels.

## 13. Regulation by POPs and COPs

In humans, pyrin-only proteins (POPs) and CARD-only proteins (COPs) regulate the inflammasome at the level of death-fold interactions. With the exception of caspase-12, POPs and COPs lack the mouse genome, which suggests humans have evolved more complex inflammasome regulatory systems [91]. The POPs include POP1 (also known as PYDC1) and POP2 (also known as PYDC2), and both inhibit PYD interactions between the receptor protein and the ASC adaptor molecule. POP1 shows a higher homology to ASC^PYD^ than POP2 and therefore inhibits inflammasome formation by sequestering ASC from other inflammasome-forming NLRs [92]. POP2 is surprisingly similar to the PYD domain of NLRP2 and NLRP7 and is thought to interact with other NLR^PYD^ proteins, thereby preventing inflammasome formation [93]. Both POP1 and POP2 can prevent NF-κB activation [92,93]. 

The COPS proteins consist of several members including CARD16 (also known as pseudo-ICE or COP1), CARD17 (also known as INCA), CARD18 (also known as ICEBERG), caspase-12s, and Nod2-S [94]. COP proteins act as decoy inhibitors and sequester procaspase-1 via CARD-CARD interactions, thereby preventing its activation in the inflammasome. For example, CARD 17 is upregulated by IFN-γ in the monocytic cell lines THP-1 and U937 and interacts with procaspase-1 to supress IL-1β processing and release in LPS-stimulated macrophages [95].

## 14. Regulation by Type I Interferons

Type I interferons restrict IL-1β production by two distinct mechanisms. Depending on the cell type, type I interferons through the STAT3 signalling pathway can induce autocrine and paracrine production of the anti-inflammatory cytokine IL-10, which inhibits the synthesis of proIL-1β and proIL-18. Additionally, type I interferons signalling through the STAT1 transcription factor can repress the activity of the NLRP1 and NLRP3 inflammasome, thereby subduing IL-1β production [96]. For the AIM2 inflammasome, *Irf3*-deficient mouse macrophages, which are unable to secrete type I interferons, have impaired AIM2 activation in response to *Francisella tularensis* infection, indicating that an intact, type I interferon response is required for AIM2 activation [75]. Interestingly, activation of the AIM2 inflammasome in response to mouse cytomegalovirus does not require an intact type I interferon response [77]. The mechanisms pertaining to the selective requirement of type I interferons for the clearance of certain infections remain unclear. 

Evidence suggests that cells of the adaptive immune response can also dampen inflammasome activation. In mouse macrophages and dendritic cells, effector CD4^+^ T cells and memory T cells suppress activation of the NLRP1 and NLRP3 inflammasomes. For the NLRP3 inflammasome, the inhibitory effect requires the cell-to cell contact and could be mimicked by macrophage stimulation with members of the TNF family such as, CD40L, OX40L, and RANKL. Interestingly, the negative feedback loop exerted by T cells is only evident for the NLRP1 and NLRP3 inflammasome, and was absent for the NLRC4 inflammasome [97]. 

## 15. Regulation from Inflammasome Components

Inflammasomes components can themselves indirectly impact inflammasome formation and IL-1β release. For example, NLRP12 acts as a negative regulator of the NF-κB pathway through its interaction and regulation of NIK and TRAF3, and dysregulation of NF-κB is associated with colonic inflammation and cancer [98]. NLRP10 interacts with ASC, even though it lacks a ligand sensing LRR, and is thought to negatively regulate the inflammasome by sequestering ASC [99,100]. The ASC adaptor protein, in addition to the full length ASC, also exists as three novel isoforms, ASC-b, ASC-c, and ASC-d. ASC-c exerted an inhibitory effect on NLRP3 inflammasome formation by only colocalise with caspase-1, and not NLRP3. ASC-d failed to colocalise with either caspase-1 or NLRP3, suggesting an undetermined function for this isoform [17]. 

Emerging evidence indicates NLRP7 is able to regulate inflammasomes; however, conflict reports argue about the nature of the negative regulation. Reconstitution experiments in HEK293 cells have shown that NLRP7 inhibits NLRP3 and caspase-1-mediated release of IL-1β, and co-immunoprecipitation studies indicated NLRP7 directly interacts with procaspase-1 and proIL-1β [101], while other work focusing on NLRP7 overexpression and gene-specific mutations have indicated that NLRP7 inhibits NF-κB activation by an unknown mechanism or inhibits release of IL-1β [102]. Positive regulation is affirmed by the formation of the NLRP7 inflammasome in response to microbial-acylated lipopeptides [81].

## 16. Inflammasome Complexes and the Intestinal Environment

Mouse models that stimulate colitis, such as DSS or DSS/azoxymethane (AOM), have provided an accessible framework for investigating the role of inflammasomes in diseases that affect the gastrointestinal tract. Differences in the experimental conditions used for colitis induction and pathogen infection have resulted in many discrepancies regarding the redundant or necessary role of individual inflammasome complexes in protecting against colitis [22].

Mice deficient in *Nlrp3*, *Nlrc4*, *IL-1β*, *Casp1/11*, and *Asc*, when challenged by DSS, have all shown increased susceptibility to colitis, disease exacerbation, frequent mortality, and increased tumor formation when compared to DSS challenged wild type mice, suggesting these components aid in colitis protection [103,104,105,106,107,108,109]. Additionally, Nlrp6 and Nlrp3 have been shown to negatively regulate colitis-associated tumorigenesis [109,110]. 

Disease exacerbation has also been a feature of DSS-challenged, *Nlrp6*-deficient mice [111]. Not reported in Nlrc4- and Nlrp3-deficient mice but associated with Nlrp6 deficiency is a reduction in the thickness of the mucus layer. The reduction in mucus has been attributed to defects in mucin granule exocytosis and reduced autophagy mechanisms in goblet cells, which suggests that, unlike NLRP3 and NLRC4, NLRP6 orchestrates downstream mechanisms involved in bacterial defences [111,112]. NLRP6 is reported to influence the composition of the microbial ecology, with *Nlrp6*, *il18*, *Asc*, and *Casp1*-deficient mice developing a colitis-forming microbiota dominated by TM7 and *Prevotellaceae* (*Bacteroidetes* phyla) species [111]; however, these results have been refuted by further work using microbial phylogenetic analyses of littermate-controlled experiments [113].

Alterations in the composition of the gut microbiota have been reported for IBD patients [114,115]. In general, UC patients exhibit higher overall bacterial counts, while in CD the bacterial counts are lower but associated with a higher proportion of unclassified *Bacteroidetes* spp. and a higher diversity of TM7 phylotyes. Increases in *Enterobacteriaceae*, adherent-invasive strains of *Escherichia coli*, and *Ruminococcus gnavus* populations, as well as a decrease in *Faecalibacterium* and *Roseburia*, have been reported for ileal CD [116,117,118]. Interestingly, disease remission in UC induces microbial populations comparable to healthy patients, while in CD the microbial population is reportedly not altered by disease remission, remaining constant in active and quiescent disease states [116].

Human work investigating the role of individual inflammasomes on the development of gastrointestinal diseases and CAC is currently lacking. It has, however, been demonstrated that the expression of inflammasome components such as *CASP1*, *IL-1β*, *IFI16*, and *AIM2* increases in active disease [119,120,121]. 

## 17. Future Direction

The dysregulation of inflammasomes and their importance in maintaining intestinal health has been demonstrated by mice deficient in inflammasome components in DSS/AOM models of intestinal inflammation. Population-based studies have identified possible risk polymorphisms associated with UC and CD. The IL-1 neutralising agent has provided a remarkable reduction in clinical symptoms for CAPS patients. Taken altogether, these findings highlight the potential therapeutic benefits of targeting individual inflammasome complexes to complement mainstream therapeutic options.

Currently, the focus of treatment for IBD patients is to induce and maintain clinical remission. This is usually achieved by a combination of antibiotics, vitamin support, immunomodulators, corticosteroids, 5-aminosalicylates, biologic therapies, and surgery [1]. For many patients, however, active disease persists despite treatment. Recently, the compound MCC950 was shown to inhibit NLRP3 non-canonical and canonical IL-1β production in both mouse bone-marrow-derived macrophages and human monocyte-derived macrophages [122]. The ability of this compound to consistently inhibit inflammasome-mediated IL-1β production provides promise for clinical trials and future therapeutic options.

While mice work has been pivotal to determining the regulatory role of inflammasomes in intestinal health, questions still remain. Firstly, what is the exact role of inflammasomes, such as the NLRP3 complex, in intestinal immune responses? Secondly, how accurately do the findings in mice translate to the mechanisms that induce colitis in humans? Future research needs to focus on individual inflammasomes complexes: how they present in active human disease, what mechanisms they influence downstream, and if blockading alleviates disease symptoms.
